# Inpatient care utilisation and expenditure associated with objective physical activity: econometric analysis of the UK Biobank

**DOI:** 10.1007/s10198-022-01487-1

**Published:** 2022-06-24

**Authors:** Leonie Heron, Mark A. Tully, Frank Kee, Ciaran O’Neill

**Affiliations:** 1 Institute of Social and Preventive Medicine (ISPM), University of Bern, Mittelstrasse 43, CH-3012 Bern, Switzerland; 2 Centre for Public Health, Queen’s University Belfast, Institute of Clinical Science, Block A, Royal Victoria Hospital, Belfast, BT12 6BA UK; 3 School of Medicine, Ulster University, Londonderry, BT48 7JL UK

**Keywords:** Healthcare costs, Physical activity, Econometric model, Accelerometer

## Abstract

**Background:**

Physical inactivity increases the risk of chronic disease and mortality. The high prevalence of physical inactivity in the UK is likely to increase financial pressure on the National Health Service. The UK Biobank Study offered an opportunity to assess the impact of physical inactivity on healthcare use and spending using individual-level data and objective measures of physical activity. The objective of this study was to assess the associations between objectively measured physical activity levels and future inpatient days and costs in adults in the UK Biobank study.

**Methods:**

We conducted an econometric analysis of the UK Biobank study, a large prospective cohort study. The participants (*n* = 86,066) were UK adults aged 43–79 who had provided sufficient valid accelerometer data. Hospital inpatient days and costs were discounted and standardised to mean monthly values per person to adjust for the variation in follow-up times. Econometric models adjusted for BMI, long-standing illness, and other sociodemographic factors.

**Results:**

Mean follow-up time for the sample was 28.11 (SD 7.65) months. Adults in the most active group experienced 0.037 fewer days per month (0.059–0.016) and 14.1% lower inpatient costs ( – £3.81 [ – £6.71 to  – £0.91] monthly inpatient costs) compared to adults in the least active group. The relationship between physical activity and inpatient costs was stronger in women compared to men and amongst those in the lowest income group compared to others. The findings remained significant across various sensitivity analyses.

**Conclusions:**

Increasing physical activity levels in the UK may reduce inpatient hospitalisations and costs, especially in women and lower-income groups.

**Supplementary Information:**

The online version contains supplementary material available at 10.1007/s10198-022-01487-1.

## Background

Physical inactivity increases the risk of several chronic diseases. [1] The prevalence of several of these conditions such as type 2 diabetes, cardiovascular disease and cancer is increasing in the United Kingdom (UK), [2] as is their financial impact on the National Health Service (NHS). Approximately 40% of UK adults are physically inactive. [3] The high prevalence of physical inactivity in the UK may increase financial pressure on the NHS. In recent years, evidence has emerged of the cost of physical inactivity in the UK and globally. [4–6]

Previous studies have used a population attributable fraction approach to estimate the percentage of healthcare spending on five diseases associated with increased risk from physical inactivity. [4–6] This ‘top down’ approach is limited by the assumptions on which it is based. Data on the relative risk and prevalence of physical inactivity are usually derived from several sources e.g., meta analyses and survey data, whereby extrapolation to the population may be open to question. Furthermore, subgroup analysis may not be possible. An econometric (‘bottom up’) approach can in principle overcome some of these issues using observed individual-level data to model the effect of physical inactivity on healthcare spending, adjusting for sociodemographic variables and known confounders. This approach avoids the assumptions of the ‘top down’ approach and facilitates comparison between population subgroups.

In addition, self-reported measures of physical activity are commonly used in epidemiological studies, partly due to the previous prohibitively high costs of accelerometers and expertise required for their analysis. Objective measures of physical activity, such as accelerometers, are subject to less recall bias and therefore may be more reliable at capturing sedentary time and light physical activity than self-reported techniques. [7, 8] In recent times, accelerometers have become a cheaper and more feasible method of measurement in large studies. For example, participants in the UK Biobank study provided physical activity data using accelerometers, which presents a welcome opportunity to assess the impact of physical activity on healthcare using an objective measure. To assess whether more active individuals subsequently spend fewer days in hospital and incur lower costs, we examined these associations in the UK Biobank cohort. We explored the relationship between objectively measured physical activity and subsequent inpatient days and costs in UK adults using individual-level prospective data. In this paper, we examine these relationships in the full sample and in samples partitioned by gender and by income level, to explore relationships among subgroups.

## Methods

### Study population

The UK Biobank is a large prospective study of 502,511 adults aged 40–69 living in England, Scotland and Wales. [9] Participants attended recruitment centres between 2006 and 2010. The study had ethical approval from the North West—Haydock Research Ethics Committee, reference 11/NW/0382. This analysis used anonymised data and therefore did not require additional ethical approval. This econometric analysis uses a healthcare payer (NHS) perspective.

### Outcome variables

#### Inpatient care utilisation

NHS primary care, hospital records from Hospital Episode Statistics (HES) in England and the Patient Episode Database (PEDW) in Wales, and death registrations, were linked with individuals in the UK Biobank. The HES records are available from 1996 to 2017 and the PEDW records from 1999 until 2016. Our analysis focused solely on inpatient activity: patients who had been referred beyond primary care whose needs were more likely to have been independently assessed thus avoiding issues of differential preferences for care. The period of follow-up was from the week during which the participant provided accelerometer data to the end of the HES/PEDW records. In order to make the inpatient data comparable, we summed the total inpatient days and divided by months of follow up. Inpatient days were discounted to reflect the time preference concept, where a hospital episode in the present year is valued differently to an episode in the future. Further detail on the inpatient episodes included can be found in Additional File 1.

#### Inpatient care costs

We monetised inpatient episodes using 2017 unit costs of health and social care from the Personal Social Services Research Unit [10] with costs for subsequent years discounted by 3.5% (see Additional File 1 for more information). Inpatient costs depended on episode type (overnight/day case and elective/non-elective) and length of stay (long/short). We divided the total costs incurred by months of follow up to create a variable of mean monthly inpatient costs.

### Explanatory variables

A sample of the UK Biobank cohort were invited to wear a wrist-worn accelerometer. Over 100,000 participants wore an accelerometer for seven days in 2013–2015. Doherty et al. calibrated the raw acceleration data to create variables of overall acceleration average in milligravities, a proxy for total physical activity energy expenditure. [11, 12] We used the average overall acceleration to estimate the physical activity level of the participants. Individuals with insufficient wear time (sufficient wear time was defined as ≥ 72 h and data recorded in each one hour period of the 24 h cycle), poorly calibrated data, or recording problems were excluded. [12]

Participants were divided into tertiles for the analysis based on their overall acceleration average: tertile 1, least active (2.6 mg to 24.0 mg); tertile 2 (24.0 mg to 30.4 mg); tertile 3, most active (30.4 mg to 224.5 mg). Acceleration in milligravities is not easily conceptualised, therefore we used tertiles. We estimated the median minutes spent in moderate to vigorous intensity physical activity, equivalent to at least brisk walking (≥ 4.5 metabolic equivalents of task [METs]) and the proportion of time spent in each physical activity state according to accelerometer data using thresholds, that had been previously estimated [13, 14] (see Additional File 1). However, since the thresholds were based on small samples of younger adults using a different accelerometer, we divided the participants into tertiles according to their average overall acceleration for the analysis. We also conducted an additional analysis using self-reported physical activity recorded at recruitment.

### Covariates

The models adjusted for the following covariates based on previous analyses: [15, 16] gender (male or female); age (continuous); self-assigned ethnic background (white British, Irish, or other ethnicity); household income (< £18,000, £18,000 to £30,999, £31,000 to £51,999, £52,000 to £100,000, > £100,000); body mass index (BMI) (< 18.5 kg/m2, 18.5–25 kg/m2, 25 to 30 kg/m2, > 30 kg/m2); waist-to-hip ratio (continuous); Townsend deprivation index (quintiles); long-standing illness, disability or infirmity (yes or no); smoking status (never, previous, or current); marital status (married/cohabiting or not); and education (university level or not). The covariates were recorded at time of recruitment in 2006–2010.

### Statistical analysis

We estimated the effect of objectively measured physical activity on inpatient care (mean monthly inpatient days) and associated costs (mean monthly inpatient costs). We used generalised linear models (GLMs), which can accommodate skewed data better than an ordinary least squares model. [17] The Akaike and Bayesian information criteria (AIC and BIC) and the Modified Park test (17) were jointly used to determine the most appropriate link function and distribution family. We also compared this model in terms of AIC and BIC with a GLM estimated on the continuous part of the two part model to provide further assurance as to its superiority in terms of fit. These results supported the model reported here; details are provided in supplementary materials.

We estimated the incremental effects of the more active tertiles (tertiles 2 and 3) on mean monthly inpatient days and costs, using the least active tertile 1 as a reference. We also estimated mean monthly inpatient days and costs for each physical activity tertile, keeping all other covariates constant in a marginal effects analysis. We calculated the percentage difference in inpatient costs, comparing the more active tertiles with the least active tertile. The models of all participants included an interaction term between age and gender. The estimates used robust standard errors. Data analysis was performed using Stata version 15.1 (StataCorp, College Station, Texas).

### Sensitivity analyses

We conducted several sensitivity analyses to assess the robustness of our findings. Firstly, we ran the main models separately for men and women to assess how the relationships might vary by gender. Then, we graphed the association between objectively measured physical activity as a continuous variable and inpatient costs at 5-year intervals of age, for men and women. Additionally, we assessed the association between accelerometer measured physical activity and mean monthly inpatient days by household income by running the main model with an interaction term between continuous acceleration and household income category.

Several additional sensitivity analyses were conducted (see Additional File 1). In summary, we explored (i) the exclusion of participants with long-standing illness; (ii) the inclusion of participants who died within two years of baseline; (iii) the inclusion of participants with less than 1 year of follow up; (iv) the inclusion of episodes of unknown type; (v) no discount applied to inpatient days; (vi) an alternative discount rate of 1.5% applied to inpatient days; (vii) potential endogeneity between BMI and physical activity, using a residual harvesting approach [18]; (viii) using self-reported physical activity as the explanatory variable; and (ix) using an alternative two-part model.

## Results

**Fig. 1:**
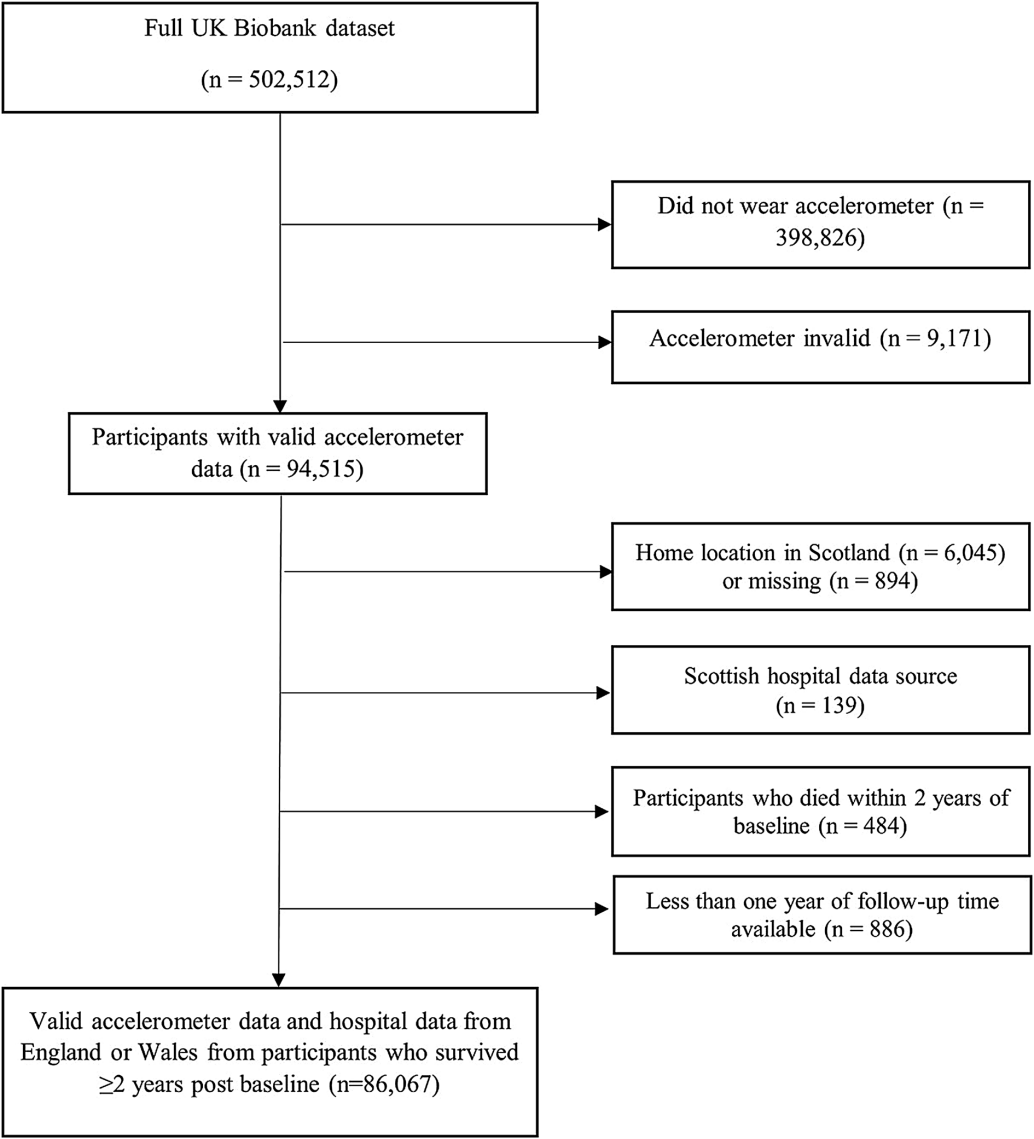
Flowchart of participant inclusion for analysis

The sample included 86,066 individuals aged 43–79 years at baseline with valid accelerometer data for analysis after excluding those who had died within two years of baseline, had insufficient follow up time, or lived in Scotland (Fig. 1). Two-thirds (67.6%) of the participants had no inpatient episodes during the follow up time (mean and median = 2 years, 4 months), resulting in a highly skewed distribution of data. The remaining third of participants incurred mean monthly inpatient costs of £92.74 (SD = £199.68). The majority of participants were female, white British, and lived with a spouse or partner (Table 1 and Table S4). Most had never smoked and self-reported high levels of physical activity, with 70% meeting national recommendations in terms of self-reported physical activity. Participants in tertile 1 achieved a median 30.2 min per week of activity at an intensity equivalent of at least brisk walking (≥ 4.5 METs), whereas tertiles 2 and 3 had higher medians of 70.6 min and 151.2 min, respectively (Table 1). Individuals with higher objective physical activity levels were more likely to be younger women with higher household incomes (Table 1). The least active tertile included relatively more people in overweight or obese BMI categories and who had long-standing illnesses, which were included as covariates in the subsequent analysis. The crude relationship between the covariates and mean monthly inpatient days and costs suggested that higher physical activity level, female gender, younger age, higher household income, normal BMI, lack of long-standing illness, not smoking, and having a university education were correlated with fewer inpatient days and lower inpatient costs (Table S3).

**Table 1:** Descriptive statistics by physical activity tertile in the UK Biobank participants

	Physical activity (accelerometer-measured)		
	Tertile 1, least physically active (*n* = 28,719)	Tertile 2 (*n* = 28,687)	Tertile 3, most physically active (*n* = 28,660)
Physical activity equivalent to ≥ 4.5 METs (mins/week), median (IQR)	30.2 (30.2)	70.6 (50.4)	151.2 (100.8)
*Gender*			
Female	14,731 (51.3%)	16,721 (58.3%)	17,084 (59.6%)
Male	13,988 (48.7%)	11,966 (41.7%)	11,576 (40.4%)
Age at baseline (years), mean (SD)	64.68 (7.41)	62.41 (7.70)	59.96 (7.69)
*Age at baseline (years)*			
40 to < 50	1,339 (4.7%)	2,237 (7.8%)	3,560 (12.4%)
50 to < 60	5,876 (20.5%)	8,117 (28.3%)	10,427 (36.4%)
60 to < 70	13,609 (47.4%)	13,172 (45.9%)	11,704 (40.8%)
70 to < 80	7,895 (27.5%)	5,161 (18.0%)	2,969 (10.4%)
*Ethnic background*			
White British	26,277 (91.9%)	26,069 (91.2%)	25,581 (89.6%)
Irish	677 (2.4%)	635 (2.2%)	647 (2.3%)
Mixed ethnicity or other ethnic group	1,650 (5.8%)	1,888 (6.6%)	2,335 (8.2%)
*Household income (before tax, £)*			
Less than 18,000	4,825 (18.9%)	3,522 (13.7%)	3,099 (12.0%)
18,000 to 30,999	6,873 (26.9%)	6,243 (24.3%)	5,659 (21.9%)
31,000 to 51,999	6,982 (27.3%)	7,447 (29.0%)	7,673 (29.7%)
52,000 to 100,000	5,456 (21.3%)	6,574 (25.6%)	7,196 (27.9%)
Greater than 100,000	1,428 (5.6%)	1,925 (7.5%)	2,196 (8.5%)
*BMI in categories*			
Underweight (< 18.5 kg/m^2^)	101 (0.4%)	125 (0.4%)	258 (0.9%)
Normal weight (18.5 to 25 kg/m^2^)	7,783 (27.2%)	11,094 (38.7%)	14,408 (50.3%)
Overweight (25 to 30 kg/m^2^)	12,333 (43.1%)	12,261 (42.8%)	10,765 (37.6%)
Obese (> 30 kg/m^2^)	8,386 (29.3%)	5,157 (18.0%)	3192 (11.2%)
Waist to hip ratio, mean (SD)	0.88 (0.09)	0.86 (.09)	0.84 (0.08)
Long-standing illness, disability or infirmity	10,521 (37.5%)	7,655 (27.2%)	5,949 (21.1%)
*Smoking status*			
Never	15,493 (54.1%)	16,497 (57.7%)	16,945 (59.3%)
Previous	10,781 (37.7%)	10,287 (35.9%)	9,974 (34.9%)
Current	2,358 (8.2%)	1,831 (6.4%)	1,666 (5.8%)
Marital status: living with husband, wife or partner	21,462 (92.1%)	21,909 (91.3%)	22,036 (90.8%)
University educated	11,531 (40.2%)	12,375 (43.1%)	12,557 (43.8%)
Follow-up time (months), mean (SD)	28.11 (7.65)	28.40 (7.68)	28.59 (7.73)

The GLMs used a log link and a Gamma distribution, as determined by information criteria and statistical tests (see Additional File 1), and adjusted for the covariates described in the methods section. Higher levels of physical activity were associated with significantly fewer inpatient days (Table 2). There was a decreasing trend in inpatient days with increased objective physical activity, evident in the decreasing number of predicted days. Participants in the middle tertile experienced 0.024 fewer inpatient days per month (95% CI  – 0.047 to  – 0.001) while the most active tertile had 0.037 fewer days (95% CI  – 0.059 to  – 0.016), equivalent to 0.3 and 0.5 fewer annual inpatient days.

**Table 2:** Inpatient days and costs by physical activity tertile in the UK Biobank participants

	Physical activity tertile		
	1 (least active)	2	3 (most active)
*Monthly inpatient days*			
Incremental effect (95% CI)*	REF	** – 0.024 (-0.047, – 0.001)**	** – 0.037 ( – 0.059, – 0.016)**
Predicted mean (95% CI)	0.165 (0.144, 0.186)	0.140 (0.123, 0.157)	0.128 (0.116, 0.140)
Predicted mean annual inpatient days^†^	2.0	1.7	1.5
*Monthly inpatient costs*			
Incremental effect (95% CI)	REF	** – £3.09 ( – £5.75, – £0.42)**	** – £3.81 ( – £6.71, – £0.91)**
Predicted mean (95% CI)	£27.37 (£25.09, £29.65)	£24.21 (£22.63, £25.78)	£23.52 (£21.67, £25.37)
Percentage difference in costs	REF	-11.5%	-14.1%

^*^Bold text indicates significance at 5%. ^†^Inpatient days per year were estimated by multiplying days per month by 12

Higher levels of objective physical activity were also associated with significantly lower inpatient costs (Table 2). The middle tertile incurred 11.5% lower costs ( – £3.09 [95% CI  – £5.75 to  – £0.42]) than the least active tertile and the most active tertile incurred 14.1% lower monthly inpatient costs ( – £3.81 [95% CI  – £6.71 to  – £0.91], equivalent to £45.72 annually). There was a negative trend between increasing objective physical activity and decreasing inpatient costs.

### Sensitivity analysis

In separate models for each gender, the associations were the same as above except for monthly costs in men only, which used a square root link (Table 3). There was a similar decreasing trend in inpatient days seen in men and women separately, although the associations were only significant in the most active group, tertile 3. The effect of physical activity on inpatient days was stronger in men. Furthermore, men had more predicted inpatient days than women in both of the more active tertiles. With respect to inpatient costs, objective physical activity was associated with significantly lower costs in women, although not in men. There was also no clear trend in the association in men. There were no clear trends or significant associations in models of inpatient days or costs when self-reported physical activity was used as the explanatory variable (see Additional File 1).

**Table 3:** Inpatient days and costs by gender and physical activity in the UK Biobank participants

	Physical activity tertile	Incremental effect on mean monthly inpatient days (95% CI)*	Incremental effect on mean monthly inpatient costs (95% CI)*
Women (*n* = 48,537)	1 (least active)	REF	REF
	2	– 0.017 ( – 0.041, 0.008)	– **£3.73 (** – **£6.97, ** – **£0.49)**
	3 (most active)	– **0.031 (** – **0.054, ** – **0.009)**	– **£5.53 (** – **£8.93, ** – **£2.13)**
Men (*n* = 37,534)	1 (least active)	REF	REF
	2	– 0.032 ( – 0.067, 0.003)	– £2.49 ( – £6.33, £1.35)
	3 (most active)	– **0.047 (** – **0.081, ** – **0.013)**	– £1.01 ( – £5.09, £3.07)

^*^Bold text indicates significance at 5%. *CI*  confidence interval

We observe that physical activity is associated with lower inpatient costs in women compared to men from ages 40 to 61 (Fig. 1 in additional file). In older age, the apparent effect of physical activity on inpatient costs is stronger for men. The confidence interval widens with increased age, however, it appears that there is a significant difference in the effect of physical activity on inpatient costs between the youngest and oldest participants.

**Fig. 2:**
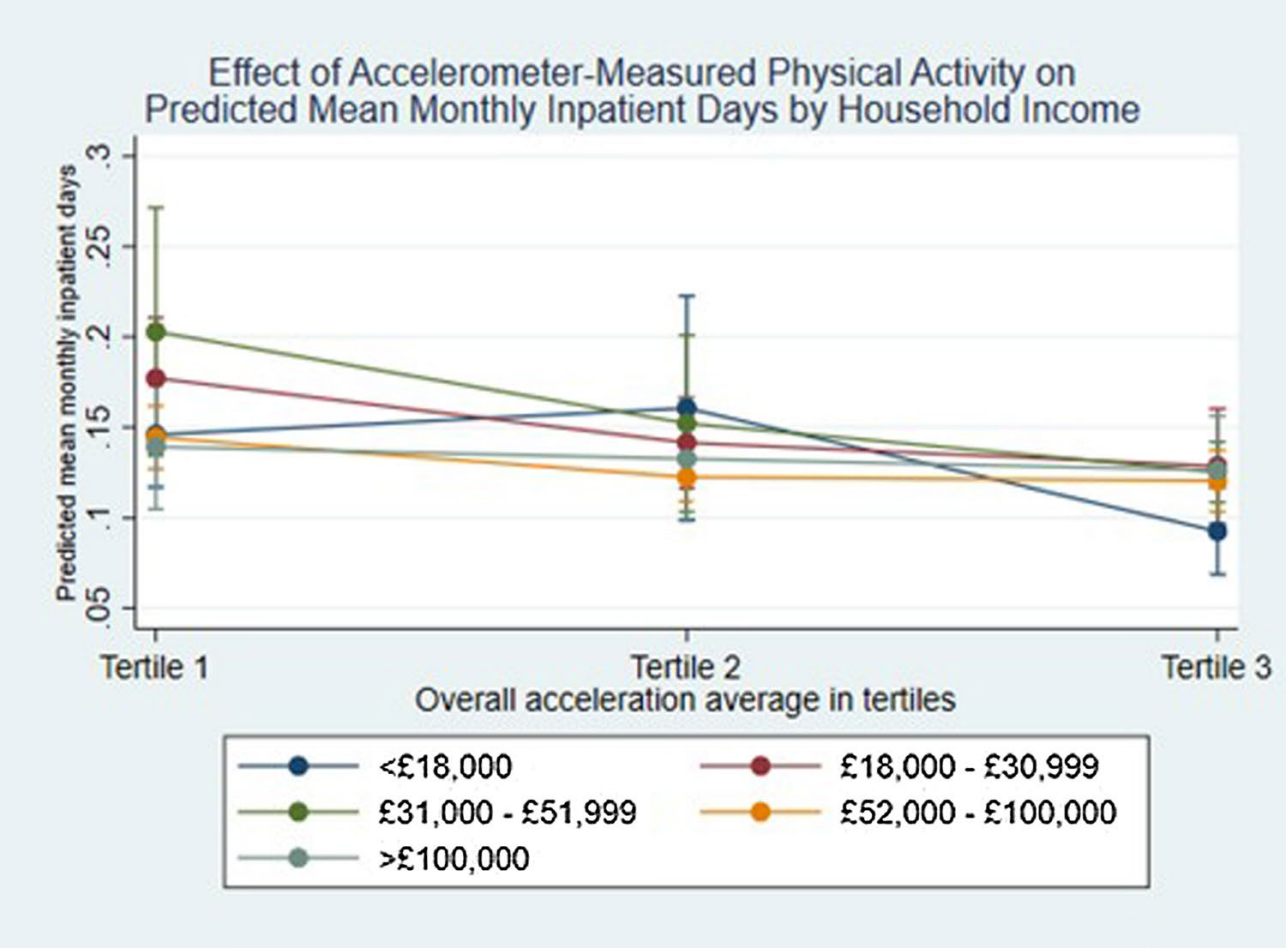
Effect of accelerometer-measured physical activity on inpatient days by household income

**Fig. 3:**
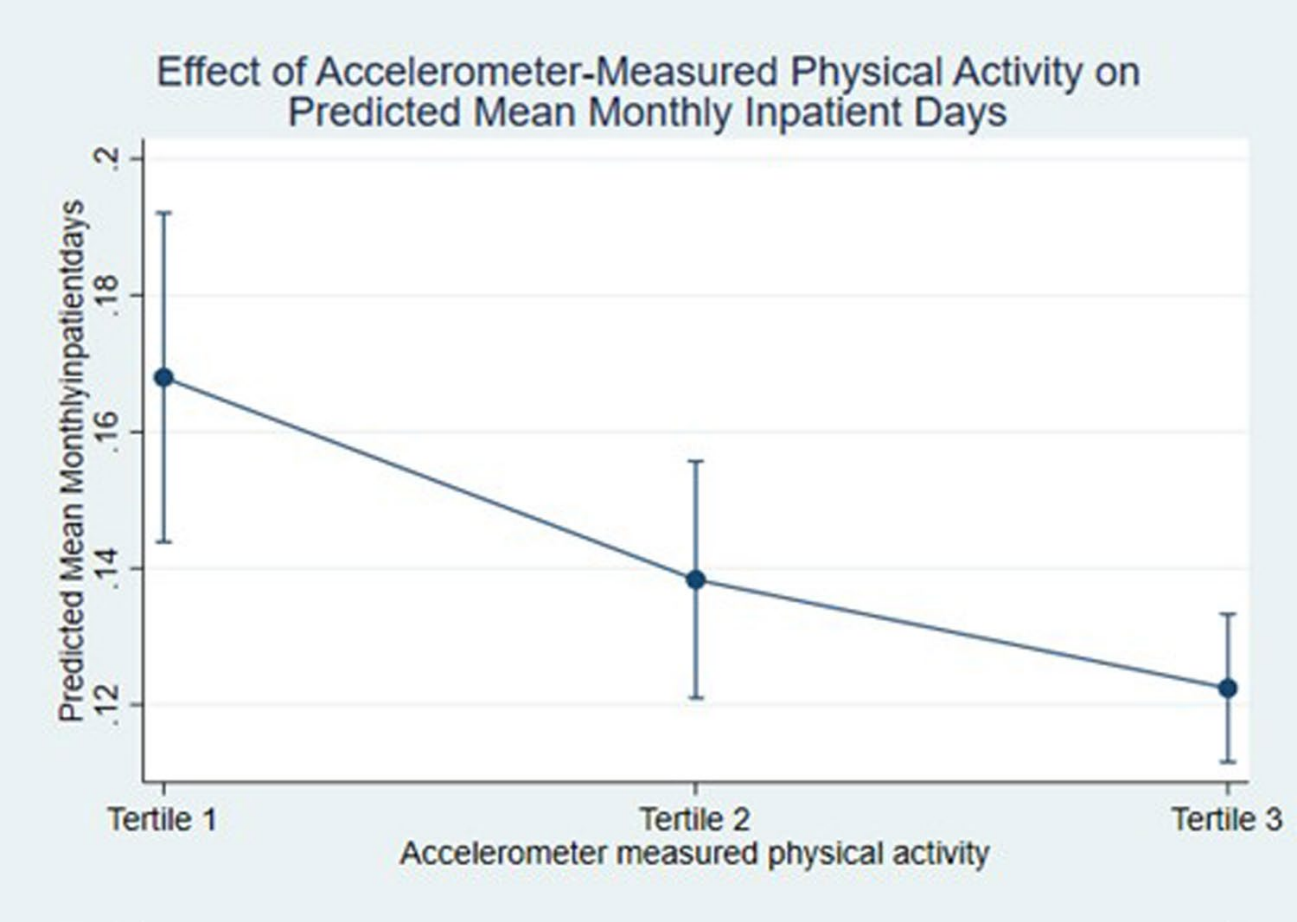
Effect of accelerometer-measured physical activity on inpatient days

The association between physical activity and inpatient days differs among household income categories (Fig. 2) and from the overall estimate shown in Fig. 3 (The highest income groups (> £52,000 per annum) experience little to no change to the number of inpatient days at different levels of physical activity. There is a non-significant trend in the middle income groups (£18,000–£51,999 p.a.) of fewer inpatient days as physical activity level increases. However, the lowest income category (< £18,000 p.a.) experience significantly fewer inpatient days in the highest tertile of physical activity compared to the least active tertile. In a series of further sensitivity analyses, the results did not materially change from the main model presented (Table S6).

## Discussion

### Summary of findings

The results of the study show that higher levels of accelerometer measured physical activity were associated with significantly fewer inpatient days and reduced inpatient costs, after adjusting for BMI, long-standing illness, and other sociodemographic factors. The more active individuals incurred lower costs than the least active group ( – £3.09/month [95% CI  – £5.75 to  – £0.42] in tertile 2 and  – £3.81/month [95% CI  – £6.71 to  – £0.91] in tertile 3). Sensitivity analyses revealed that higher physical activity reduced inpatient days in men and women, but only reduced inpatient costs in women. Inpatient costs reflect the intensity of care as well as length of stay, indicating that physical activity reduced the need for more intense inpatient care in women. The most active participants in the lowest income group experienced the most dramatic reduction in inpatient days, in stark contrast to the highest income group in which physical activity appeared to have no effect.

### Strengths and limitations of the study

Strengths of the study include its large analytic sample and the use of accelerometer data, which was sufficient to identify small differences between individuals and less susceptible to the bias attendant on self-reported data. [8] NHS inpatient records were available for all participants. Nevertheless, the analysis was limited by several factors. Costs of primary care, prescriptions, private care, and out-of-pocket costs were excluded from the analysis due to data limitations. We expect inpatient care to be correlated with other healthcare use, although inpatient care usually indicates more severe health status. The cohort includes more women, less ethnic minorities and reported higher incomes and levels of education compared to the UK population, therefore it is not perfectly representative of the general population. [19] The lack of representativeness may limit the study’s external validity although Stamatakis et al. reported that the association between physical activity and health outcomes was minimally affected by this issue. [20]

### Comparison with similar studies

Our results are in line with other econometric studies, which also found that higher levels of physical activity were associated with lower healthcare use or costs in population based samples [15, 16, 21, 22]. Ku et al. and Carlson et al. also reported similar percentage differences in healthcare expenditure. [16, 22] Karl et al. used objective measures of physical activity in addition to self-reported physical activity, although the accelerometer data were only available for a subsample. [16] The authors reported that the accelerometer measured physical activity affected healthcare costs but no effect was found for the association with self-reported physical activity in line with our findings, in a comparable sample of European adults aged 48 to 68 years old. To the authors’ knowledge, this is the first study to assess the relationship between objective physical activity and healthcare use and costs using econometric methods in a UK population.

### Implications

Physical inactivity could be related to other potential economic costs such as reduced productivity and absenteeism due to morbidity or mortality. Future studies could explore the associations between level of physical activity and other healthcare use and costs in a wider range of age groups, with a focus on lower income groups since they experienced the strongest effect of physical activity. Furthermore, adopting a compositional analysis approach should be considered [23] for we recognise that sleep duration and quality has not been included in our models and different subgroups of the population may substitute domains of physical activity in different ways and both may impact costs.

## Conclusion

Our findings highlight the importance of maintaining high levels of physical activity to reduce risk of disease and subsequent healthcare use. The effect was stronger in the lowest income groups and in women. Increasing physical activity levels in the UK populations could reduce the burden on the NHS in terms of hospital inpatient care.

## Supplementary Information

Below is the link to the electronic supplementary material.

Supplementary file1 (DOCX 214 KB)

## Data Availability

The data that support the findings of this study are available from UK Biobank project site, subject to successful registration and application process. Further details can be found at https://www.ukbiobank.ac.uk/.

## Abbreviations

UK: United Kingdom
NHS: National Health Service
HES: Hospital Episode Statistics
PEDW: Patient Episode Database
METs: Metabolic equivalents of task
BMI: Body mass index
GLM: Generalised linear models
AIC: Akaike information criteria
BIC: Bayesian information criteria
CI: Confidence interval
