# Supplementary material for: Inpatient care utilisation and expenditure associated with objective physical activity: econometric analysis of the UK Biobank
Source: Eur J Health Econ. 2022 Jun 24;24(4):489–97. doi: 10.1007/s10198-022-01487-1 (PMC10175475; doi:10.1007/s10198-022-01487-1)
Supplement: Supplementary file 1 — Supplementary file1 (DOCX 214 KB) [file 10198_2022_1487_MOESM1_ESM.docx]

**Supplementary Material**

**INPATIENT HOSPITAL EPISODES**

We removed any inpatient hospital episodes that occurred before baseline to assess the prospective effect of physical activity, measured between June 2013 and December 2015. Participants resident in Scotland or with Scottish health records were excluded from the analysis since the Scottish health data were not comparable with English and Welsh records.

An inpatient is admitted to hospital for treatment and occupies a hospital bed, without necessarily spending the night. A hospital episode is the time during which a patient is under the care of one consultant, and a hospital admission may consist of one or more episodes. The data available included the type of episode (general, maternity, or psychiatric); the type of admission (elective or non-elective); and the dates of admission and discharge. Maternity episodes, considered distinct to service use related to ill health, or those with missing episode type were removed from analysis. There were no psychiatric episodes in the sample. Length of follow up ranged from 1 year to 3 years 10 months. Participants with insufficient follow up time to provide robust estimates of service use (less than one year) were excluded. To make the inpatient data comparable, the days spent in hospital as an inpatient were summed and divided by months of follow-up to create a variable of mean monthly inpatient days. Inpatient days were discounted to reflect their present value in 2013 using the same methods as inpatient costs (see below) to reflect the time preference concept, where a hospital episode in the present year is valued differently to an episode in the future.

**Unit costsa**

We monetised inpatient episodes using 2017 unit costs of health and social care from the Personal Social Services Research Unit (PSSRU) (Curtis and Burns, 2017). We expressed as a discounted stream of costs arising after baseline, with the years subsequent to 2013 discounted by 3.5% (Table 1). The cost applied to each episode depended on whether it was a day case or the patient stayed overnight. If an overnight case, the cost depended on the episode type: elective/non-elective and short/long stay. A long stay was defined as one that lasted at least 21 days, as defined in National Health Service (NHS) literature (<https://improvement.nhs.uk/documents/3005/Long-stay_patients_methodology.pdf>). We summed the expenditure for all episodes experienced by each participant, and divided by the months of follow-up.

Table 1. Unit costs for inpatient hospital episodes

| Year | Average cost per episode | | | Average cost per day case |
| --- | --- | --- | --- | --- |
|  | Elective inpatient stays | Non-elective inpatient stays (long stays†) | Non-elective inpatient stays (short stays†) |  |
| 2013 | £3,903.00 | £2,953.00 | £628.00 | £727.00 |
| 2014 | £3,771.01 | £2,853.14 | £606.76 | £702.42 |
| 2015 | £3,643.49 | £2,756.66 | £586.24 | £678.66 |
| 2016 | £3,520.28 | £2,663.44 | £566.42 | £655.71 |
| 2017 | £3,401.24 | £2,573.37 | £547.27 | £633.54 |
| *2017 Unit costs from the Personal Social Services Research Unit (PSSRU) (Curtis and Burns, 2017). Costs represent the present value of the discounted stream of costs arising after baseline. Costs arising after 2013 were discounted by 3.5%.  †Long stays were defined as an episode which lasted 21 days or more, as defined in an NHS report (NHS England and NHS Improvement, 2018). | | | | |

**MISSING DATA**

Table 2. Missing data for outcomes, exposures, and covariates

| Variable | Missing observations | |
| --- | --- | --- |
|  | **N** | **%** |
| Gender | 0 | 0.00 |
| Age | 0 | 0.00 |
| Ethnic background | 42 | 0.05 |
| Household income | 643 | 0.75% |
| Body mass index | 203 | 0.24% |
| Long-standing illness | 42 | 0.05% |
| Smoking status | 42 | 0.05% |
| Marital status | 14,337 | 16.7% |
| University education | 546 | 0.63% |

**ACCELEROMETER DATA**

Doherty et al. cleaned and processed the raw acceleration data, producing a variable of overall acceleration average in milli-gravities. We removed participants with insufficient wear time or poor calibration as recommended. Participants were divided into tertiles based on their overall acceleration average: tertile 1, least active (2.6mg to 24.0mg); tertile 2 (24.0mg to 30.4mg); tertile 3, most active (30.4mg to 224.5mg). To translate the milli-gravities into better known levels of activity, we described the tertiles in terms of median minutes of activity of at least brisk walking (4.3 METs)(Ainsworth et al., 2011), as was done in a recent study of the UK Biobank (Chudasama et al., 2019). Brisk walking at 4.3 METs is equivalent to acceleration of at least 250mg, according to regression equations from a calibration study (Hildebrand et al., 2014). Hildebrand et al. assessed acceleration while a sample of 30 adults (aged 18 to 65 years) completed a range of activities wearing a comparable accelerometer on their wrists. Brisk walking is helpful in understanding level of activity since it is commonly used in PA guidelines. Therefore, we estimated time spent doing at least 250mg of acceleration. Tertile 1 had a median of 30.2 mins (IQR: 30.2) of at least brisk walking per week. Tertiles 2 and 3 had medians of 70.6 mins (IQR: 50.4) and 151.2 mins (IQR: 100.8), respectively.

We also attempted to translate the acceleration data into time spent in physical activity (PA) states (sedentary, light-intensity, moderate-intensity, and vigorous-intensity PA) using threshold cut-offs estimated by Hildebrand et al. (2014, 2017). The cut-offs were <45.8mg for sedentary behaviour; 45.8mg-93.2mg for light PA; 93.2mg-418.3mg for moderate PA; and >418.3mg for vigorous PA. We used the closest available cut-off point in the data to estimate the distribution of time the participants spent in each state (Table 5). The cut-offs are the best available for the accelerometer used and population in the UK Biobank. However, there were some limitations. Hildebrand used small samples of younger adults to calculate the cut-off thresholds. Therefore, it is likely that they do not accurately reflect the true proportion of time spent in each PA state. Nevertheless, they provide an interesting approximation and opportunity to compare the PA tertiles.

Table 3 shows the estimated time per week spent in each PA state according to tertile. The more active tertiles spend less time in sedentary behaviour and light PA states, and more time in a MVPA state.

Table 3. Estimated time spent in each physical activity state by acceleration tertile.

|  | Mean time spent in activity per week | | | |
| --- | --- | --- | --- | --- |
| Physical activity | **Sedentary behaviour** | **Light PA** | **Moderate PA** | **Vigorous PA** |
| Tertile 1, least active | 130h29m | 30h5m | 7h18m | 8m |
| Tertile 2 | 120h19m | 35h29 | 11h53m | 19m |
| Tertile 3, most active | 111h23m | 38h | 17h42m | 55m |

**INFORMATION CRITERIA AND STATISTICAL TESTS**

**Table 4. Akaike and Bayesian information criteria**

| Model |  | Model for monthly inpatient days | | Model for monthly inpatient costs | |
| --- | --- | --- | --- | --- | --- |
| Family | **Distribution** | **AIC** | **BIC** | **AIC** | **BIC** |
| Log | Gamma | -124307* | -124126* | 400344* | 400527* |
| Square root | Gamma | -124010 | -123829 | 400439 | 400622 |
| Log | Gaussian | 20761 | 20926 | 527534 | 527717 |
| Square root | Gaussian | 24360 | 24541 | 527613 | 527796 |
| Log | Poisson | 10036 | 10217 | 34,700,000 | 34,700,000 |
| Square root | Poisson | 10072 | 10253 | 34,800,000 | 34,800,000 |
| *Indicates chosen model | | | | | |

**Table 5. Modified Park Test**

|  | Model for monthly inpatient days | Model for monthly inpatient costs |
| --- | --- | --- |
| xbetahat coefficient | 1.678 | 1.638 |

**CRUDE RELATIONSHIP BETWEEN COVARIATES AND INPATIENT DAYS AND COSTS**

Table 6. Crude relationship between covariates and mean monthly inpatient days and costs

| Covariate | | Mean monthly inpatient days | Mean monthly inpatient costs (£) |
| --- | --- | --- | --- |
| Objective physical activity level | Tertile 1 | 0.083 | 40.22 |
|  | Tertile 2 | 0.051 | 27.94 |
|  | Tertile 3 | 0.037 | 21.88 |
| Gender | Female | 0.051 | 27.84 |
|  | Male | 0.066 | 32.85 |
| Age at baseline | 40-50 | 0.027 | 15.11 |
|  | 50-60 | 0.039 | 21.48 |
|  | 60-70 | 0.060 | 32.24 |
|  | 70-80 | 0.092 | 44.36 |
| Ethnic background | British | 0.058 | 30.19 |
|  | Irish | 0.055 | 29.18 |
|  | Mixed ethnicity/other | 0.051 | 28.07 |
| Household income | Prefer not to answer | 0.058 | 31.82 |
|  | Do not know | 0.074 | 37.42 |
|  | <£18,000 | 0.084 | 39.34 |
|  | £18,000 - £30,999 | 0.067 | 33.93 |
|  | £31,000 - £51,999 | 0.055 | 28.73 |
|  | £52,000 - £100,000 | 0.039 | 22.95 |
|  | >£100,000 | 0.036 | 21.26 |
| Body mass index | Underweight | 0.076 | 42.57 |
|  | Normal weight | 0.046 | 24.32 |
|  | Overweight | 0.057 | 29.53 |
|  | Obese | 0.080 | 41.78 |
| Long-standing illness | Yes | 0.096 | 47.37 |
|  | No | 0.042 | 22.87 |
| Smoking status | Never | 0.051 | 26.87 |
|  | Previous | 0.066 | 34.67 |
|  | Current | 0.066 | 31.73 |
| Marital status | Married or living with a partner | 0.054 | 29.15 |
|  | Not married or living with a partner | 0.055 | 30.00 |
| University education | Yes | 0.050 | 26.04 |
|  | No | 0.063 | 32.95 |

**ADDITIONAL SENSITIVITY ANALYSES**

**Methods**

Several additional sensitivity analyses were conducted. (1) Participants with long-standing illness were under-represented in the more active physical activity tertiles therefore they were excluded to explore whether the effect of physical activity on inpatient days was similar in the healthier participants. (2) Participants who had died within two years of baseline were re-included; they had been excluded to mitigate potential reverse causation. (3) Participants with less than one year of follow-up were re-included. (4) Episodes of unknown type were re-included and monetised using the methodology described in section 2.2.2. (5) Inpatient days were not discounted. (6) Inpatient days were discounted using an alternative discount rate of 1.5%.

(7) Endogeneity can occur when the error term in a model is correlated with an explanatory variable and can produce inconsistent results. Since BMI and physical activity are correlated, there is a risk of endogeneity. The residuals were obtained from a regression between BMI and the continuous objective physical activity variable (average overall acceleration). The residuals represent BMI which is not associated with the level of physical activity. The BMI residuals were input into the main model using methodology outlined by Terza, Basu and Rathouz (2008).

(8) For the sake of comparison, the model used self-reported physical activity recorded at recruitment as an explanatory variable in an additional analysis. The electronic questionnaire at recruitment included an adapted version of the International Physical Activity Questionnaire (IPAQ) (Craig et al., 2003). Participants provided frequency and duration of walking, moderate physical activity and vigorous physical activity. Their responses were processed and scored using estimated METs for each activity: 2.3 METs for walking, 3.0 METs for moderate activity, and 7.0 METs for vigorous activity (Donnell et al., 2020). The total estimated METs per week was used to create a binary variable which indicated whether participants achieved the equivalent of 150 minutes of walking or moderate physical activity or 75 minutes of vigorous physical activity per week, the current WHO physical activity recommendations (World Health Organisation, 2010). (9) We used a two-step model using a probit model and a generalized linear model (GLM) with a log link and gamma distribution.

**Results**

Table 6 presents the results of the additional sensitivity analyses: (1) Participants with long-standing illness excluded; (2) Participants who died within two years of baseline included; (3) Participants with less than one year of follow-up included; (4) Episodes of unknown type included; (5) Discounting inpatient days; (6) Discounting of 1.5% applied; (7) Potential endogeneity due to BMI addressed by using residuals rather than raw variable; (8) Self-reported physical activity as the explanatory variable; (9) Using an alternative two-part model

The associations did not change in models (2) and (3) which suggests that the main model is robust although only a small number of participants were re-included in these cases. Similarly, the association in (7) did not change, indicating that the model is robust with respect to the relationship between inpatient days and physical activity level. The effect was attenuated slightly in models (1) when participants with long-standing illness were excluded and (4) episodes without a type recorded were included. The effect sizes were smaller when inpatient days were not discounted in the model (5). The effect sizes were slightly smaller when inpatient days were discounted with an alternative discount rate of 1.5%, although the direction of the effect did not change in (6). There were no clear trends or significant associations in both inpatient days and costs when self-reported physical activity was used as the explanatory variable in (8). Finally, the effect sizes were slightly smaller for inpatient days and larger for inpatient costs in the two-step model, although the direction of the effect did not change in (9). We tested the family and link function for the second stage of the two-part model to confirm that log link and gamma family were more appropriate with lower AIC and BIC values. We also compared this model against the main model presented in the article using the AIC value to confirm that the model presented in the article was a better fit.

Table 7. Additional sensitivity analyses

| **Model** | **Physical activity tertile** | **Incremental effects on monthly inpatient days** | | **Incremental effects on monthly inpatient costs** |
| --- | --- | --- | --- | --- |
|  |  | **n** | **All participants** |  |
| **Main results for comparison** | 1 (least active.) | 86,066 | REF | REF |
|  | 2 |  | **-0.024 (-0.047, -0.001)** | **-£3.09 (-£5.75, -£0.42)** |
|  | 3 (most active) |  | **-0.037 (-0.059, -0.016)** | **-£3.81 (-£6.71, -£0.91)** |
| **(1) Excluding participants with long-term illness** | 1 (least active.) | 61,941 | REF | REF |
|  | 2 |  | **-0.022 (-0.041, -0.003)** | **-£3.75 (-£6.11, -£1.39)** |
|  | 3 (most active) |  | **-0.024 (-0.041, -0.006)** | **-£2.81 (-£5.42, -£0.20)** |
| **(2) Including participants who died within 2 years of baseline** | 1 (least active.) | 86,588 | REF | REF |
|  | 2 |  | **-0.025 (-0.047, -0.002)** | **-£3.26 (-£5.93, -£0.59)** |
|  | 3 (most active) |  | **-0.038 (-0.059, -0.017)** | **-£3.90 (-£6.80, -£1.00)** |
| **(3) Including participants with <1-year follow-up** | 1 (least active.) | 86,952 | REF | REF |
|  | 2 |  | **-0.025 (-0.047, -0.002)** | **-£3.00 (-£5.64, -£0.36)** |
|  | 3 (most active) |  | **-0.038 (-0.059, -0.017)** | **-£3.70 (-£6.58, -£0.83)** |
| **(4) Including episodes with type missing** | 1 (least active.) | 86,066 | REF | REF |
|  | 2 |  | -0.007 (-0.14, 0.000) | **-£2.88 (-£5.61, -£0.15)** |
|  | 3 (most active) |  | **-0.013 (-0.020, -0.006)** | **-£4.07 (-£7.03, -£1.10)** |
| **(5) Without discounting applied on inpatient days** | 1 (least active.) | 86,066 | REF | - |
|  | 2 |  | -0.025 (-0.049, -0.001) | - |
|  | 3 (most active) |  | **-0.039 (-0.062, -0.017)** | **-** |
| **(6) Discounting of 1.5% applied** | 1 (least active.) | 86,066 | REF | - |
|  | 2 |  | **-0.007 (-0.014, -0.001)** | - |
|  | 3 (most active) |  | **-0.011 (-0.017, -0.004)** | **-** |
| **(7) Residuals replacing BMI variable** | 1 (least active.) | 86,066 | REF | REF |
|  | 2 |  | **-0.023 (-0.046, -0.000)** | **-£3.33 (-£5.97, -£0.69)** |
|  | 3 (most active) |  | **-0.036 (-0.057, -0.016)** | **-£4.49 (-£7.37, -£1.60)** |
| **(8) Using self-reported PA as explanatory variable** | low | 73,016 | REF | REF |
|  | moderate |  | 0.003 (-0.005, 0.010) | £0.86 (-£2.01, £3.73) |
|  | high |  | 0.004 (-0.003, 0.010) | £2.91 (£-0.11, £5.94) |
| (9) Using an alternative two-part model | 1 (least active.) | 86,066 | REF | REF |
|  | 2 |  | **-0.007 (-0.015, -0.000)** | **-£3.34 (-£5.96, -£0.73)** |
|  | 3 (most active) |  | **-0.013 (-0.020, -0.006)** | **-£4.63 (-£7.44, -£1.81 )** |

Figure 1. Incremental effects of physical activity by age for men and women


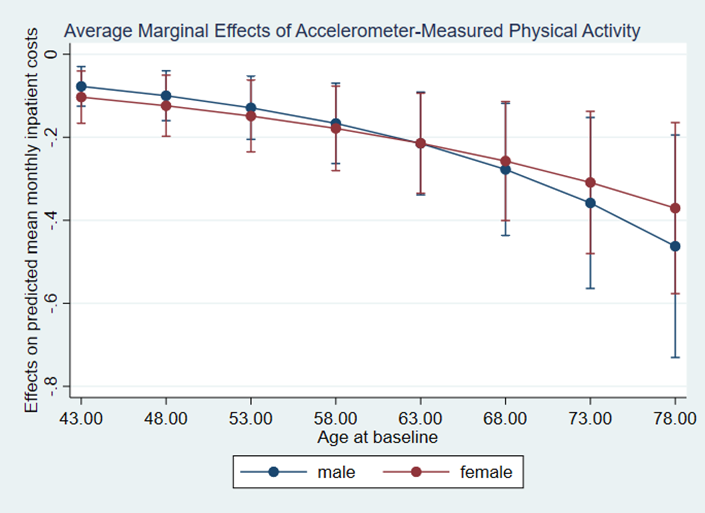


**REFERENCES**

Ainsworth, B. E. et al. (2011). 2011 compendium of physical activities: A second update of codes and MET values. *Med Sci Sport Exerc*, 43 (8), pp.1575–1581.

Chudasama, Y. V. et al. (2019). Physical activity, multimorbidity, and life expectancy: A UK Biobank longitudinal study. *BMC Med.*, 17 (1), p.108.

Craig, C. L. et al. (2003). International physical activity questionnaire: 12-Country reliability and validity. *Med Sci Sport Exerc*, 35 (8), pp.1381–1395.

Curtis, L. and Burns, A. (2017). *Unit Costs of Health and Social Care 2017*. Canterbury.

Donnell, J. O. et al. (2020). Self-reported and objectively measured physical activity in people with and without chronic heart failure: UK Biobank analysis. *Open Heart.*, 7 (1), p.e001099.

Hildebrand, M. et al. (2014). Age group comparability of raw accelerometer output from wrist-and hip-worn monitors. *Med Sci Sports Exerc*, 46 (9), pp.1816–1824.

Hildebrand, M. et al. (2017). Evaluation of raw acceleration sedentary thresholds in children and adults. *Scand J Med Sci Sports*, 27 (12), pp.1814–1823.

NHS England and NHS Improvement. (2018). *Long-stay patients methodology*.

Terza, J. V., Basu, A. and Rathouz, P. J. (2008). Two-Stage Residual Inclusion Estimation: Addressing Endogeneity in Health Econometric Modeling. *J Health Econ.*, 27 (3), pp.531–543.

World Health Organisation. (2010). *Global recommendations on physical activity for health*. Geneva.
